# Inferential talk between teachers and children across play types: a categorization based on the play continuum

**DOI:** 10.3389/fpsyg.2026.1701414

**Published:** 2026-07-03

**Authors:** Shuaili Song, Yijun Hao

**Affiliations:** 1 Affiliated Kindergarten of Hangzhou Qianjiang Foreign Language Experimental School, Hangzhou, China; 2 College of Child Development and Education, Zhejiang Normal University, Jinhua, China

**Keywords:** early childhood development, inferential talk, play, preschools, teacher-child interactions

## Abstract

Verbal interactions between teachers and children play a significant role in child development. This study systematically examined the linguistic features and bidirectional dynamics of inferential talk in teacher-child dyads across three play contexts—free play, guided play, and teacher-directed play—based on 54 Chinese preschool play activities categorized according to the play continuum framework. Participants were 41 teachers and up to 637 children from their classroom aged 3–6. By employing a four-level coding scheme of abstraction (i.e., a literal-to-inferential discourse continuum from Level 1 to Level 4), the study revealed significant differences in the level of inferencing in teacher-child language across different play types. Moreover, a significant alignment was found between the level of children’s responses and teachers’ initiating utterances, as well as between teachers’ responses and children’s initiations. Each play context displayed distinct bidirectional dynamics: free play was characterized by low-level reciprocity, guided play by matched-level engagement, and teacher-directed play by progressive scaffolding that elevated cognitive complexity. These findings affirm that play types situated along the play continuum modulate cognitive tension through differential distributions of control. The study offers empirical linguistic evidence to support the optimization of multimodal play practices and the enhancement of dialogic quality in early childhood educational settings.

## 1 Introduction

The quality of teacher-child interactions is a well-established determinant of child development outcomes (Chen and Liu, 2023; Melhuish et al., 2016). Within early childhood education settings, these interactions are predominantly mediated through language (Han et al., 2019). A critical dimension of the research concerning teacher-child verbal interaction focuses on inferential talk which generally refers to talk that emphasizes predicting future events, formulating hypotheses, and inferring causal relationships (van Kleeck et al., 1997). This type of talk corresponds to higher cognitive demands (Sigel, 1986). Empirical research demonstrates that infants as young as 8 months old are aware of causal relationships between objective phenomena (Moshman, 2004). However, it is not until around the age of two that inferential thinking begins to manifest in children’s language, at which point their comprehension and use of inferential language become directly observable (Sobel and Kirkham, 2006). Further research indicates that the use of inferential language can foster the development of vocabulary (Gonzalez et al., 2014; Tompkins et al., 2017; Mascareño et al., 2016), abstract language (van Kleeck et al., 1997), intelligence (Feldman et al., 2005), reading comprehension (Lynch et al., 2008; Oakhill and Cain, 2007; Serpell et al., 2005), social cognition (Hadley et al., 2020), mathematical reasoning (Flynn et al., 2020; Ribner et al., 2020), and scientific thinking (Booth et al., 2020) in young children.

Existing research on teacher-child inferential talk has primarily examined structured contexts such as book reading and direct instruction (Brinchmann et al., 2023; Massey et al., 2008; Tompkins et al., 2013), with comparatively limited investigation of play-based interactions. Yet emerging evidence suggests that play characterized by high language density in teacher-child exchanges (Brinchmann et al., 2023; Tamis-LeMonda et al., 2017) may constitute a uniquely productive context for advanced dialogic engagement (Hao and Hu, 2021; Tompkins et al., 2013). It has been evident that play-based interactions naturally facilitate abstract thematic discussions, particularly through pretend play scenarios that inherently require hypothesis generation and testing (Weisberg et al., 2013). Under this play context teachers are able to strategically introduce advanced lexicon and complex syntax through role-embedded modeling (Hao and Hu, 2021), with vocabulary complexity measures showing 28% higher diversity than classroom instruction (Brinchmann et al., 2023). It might be that underlying teacher-child interactions in these play settings there exists joint attention between them. When teachers and children co-construct narratives around shared play topics and plot development driven by roles, their dialogues demonstrate longer conversational turns^1^ (Tamis-LeMonda et al., 2017), more bidirectional communication (Røe-Indregård et al., 2024) and greater conducive to responsive interactions, which demonstrate the strongest and most extensive predictive power for the advancement of children’s vocabulary levels (Hadley and Dickinson, 2019; Toub et al., 2018) compared to structured instructions (such as book reading settings). The empirical evidence positions play not merely as an alternative setting, but as a pedagogically superior medium for cultivating advanced inferential discourse.

Amid the global shift toward play-based learning in preschool curricula (Baker, 2014; Gananathan, 2011; Hegde et al., 2014; Kim, 2004; Ling-Yin, 2006; Lynch, 2014; Pan and Li, 2012; Synodi, 2010), play in preschool has transcended the traditional dichotomous view that pits it against direct instruction. A more accurate model conceptualizes them as an integrated continuum. Along this continuum, agency and control shift gradually from the child to the teacher, representing a progression from free play to learning through games (Pyle and Danniels, 2016). Given the substantial variations in power dynamics and interaction patterns between teachers and children across different play types, verbal interactions manifest in distinct forms. A singular focus on any one play modality (e.g., exclusively free play or teacher-directed play) inherently limits our understanding of how inferential talk specifically operates within play contexts. Specifically, teacher-directed play may predominantly employ open-ended questioning (e.g., “Why does.?” or “What if.?”) to stimulate higher-order discussions, fostering explanatory reasoning through adult scaffolding. Free play, by contrast, more naturally could elicit imaginative language and fictional dialogue generation, serving as a catalyst for counterfactual reasoning. However, dichotomizing play into merely teacher-directed play versus free play oversimplifies the spectrum of inferential talk possibilities. Recent years have seen growing research on “guided play” as an intermediate approach. However, most studies have focused on its impact on children’s development in areas such as shaping recognition (Fisher et al., 2013), science conceptual understanding (Sliogeris and Almeida, 2019), self-regulation (Cavanaugh et al., 2017; Pyle et al., 2022), and literacy (Pyle et al., 2024). In the realm of teacher-child verbal interaction, research has primarily examined quantitative linguistic features, such as the frequency of math-related dialogue (Eason and Ramani, 2020) or the use of spatial vocabulary (Skene et al., 2022), while the qualitative aspects of dialogue and interaction patterns remain largely unexplored. Grounded in the concept of the play continuum, this study systematically reviews the research on preschool play and adopts a three-category framework—free play, guided play, and teacher-directed play. It empirically examines the patterns of teacher-child inferential talk across these three contexts and provides a micro-level linguistic analysis of real-time interactive dynamics under devious everyday play settings.

## 2 Teacher-child inferential talk in preschool everyday settings

Individual language production, when depending upon Cognitive Distancing Theory, can be characterized as existing along a literal-inferential continuum, encompassing both contextually grounded communication (e.g., labeling present objects or describing immediate surroundings) and cognitively sophisticated discourse involving abstraction (e.g., forecasting future scenarios, generating hypotheses, and establishing causal connections) (Sigel, 1986; van Kleeck et al., 1997). The construct of inferential talk is conceptually equivalent to several related terms or interpretations, including abstract language, decontextualized discourse, displaced communication, non-immediate talk, cognitively demanding speech, and high-level language (Tompkins et al., 2013). As communicative contexts shift, so too do participants’ social roles, interactional goals, and consequently, their linguistic patterning (Dickinson et al., 2014). A substantial body of research has systematically examined teacher-child inferential talk mainly across the preschool educational contexts including structured instruction and shared book reading (Chen and Liang, 2017; Danis et al., 2000; Fei et al., 2020; Hindman et al., 2012; Jin, 2014; Kucherenko et al., 2023; Liu, 2023; Zhang, 2023; Zucker et al., 2010).

In the context of instructional activities, research findings have yielded mixed results: some studies have found that teachers’ questions tend to be more literal (Fei et al., 2020; Chen and Liang, 2017), while others have reported a relatively balanced distribution between literal and inferential questioning (Jin, 2014). However, regarding children’s verbal output, a greater consensus emerges across studies, indicating that children’s literal responses generally outnumber their inferential responses (Chen and Liang, 2017; Fei et al., 2020; Jin, 2014). The conclusions become more complex in the context of shared picture book reading. Some studies have shown that teachers’ inferential questions significantly dominate (Hindman et al., 2008; Kucherenko et al., 2023; Liu, 2023; Zhang, 2023), whereas others have found no significant difference (Zucker et al., 2010). Correspondingly, children’s responses exhibit a similarly inconsistent pattern, with some studies indicating a predominance of literal responses (Danis et al., 2000) and others reporting no significant difference (Kucherenko et al., 2023; Liu, 2023; Zhang, 2023).

A body of research has additionally explored the characteristics and patterns of inferential discourse between teachers and children during play activities. Notwithstanding the relatively infrequent occurrence of teachers’ inferential language, particularly question-based inferential talk during child-directed free play (Massey et al., 2008; Zhao, 2006), and the comparatively lower frequency of decontextualized language exchanges relative to highly structured activities like book reading, mathematics, and science (Brinchmann et al., 2023; Chaparro-Moreno et al., 2022; Gest et al., 2006; Massey et al., 2008), distinct patterns emerge when examining specific play modalities. For instance, in teacher-involved pretend play scenarios, educators demonstrate a relatively balanced distribution between literal and inferential questioning, while children’s responses show a slight predominance of literal over inferential language (Tompkins et al., 2013). Similarly, during structured toy play, teachers employ marginally more decontextualized than contextualized language, with children’s verbal output maintaining relative equilibrium between these discourse types (Brinchmann et al., 2023). Existing research confirms that in open-ended free play contexts, teachers provide limited vocabulary-learning opportunities that incorporate inferential language. Paradoxically, the most frequent and linguistically rich teacher-child interactions occur during teacher-directed whole-group activities (e.g., Vitiello et al., 2012), where the potential for high-quality discourse is significantly greater (Cabell et al., 2013; Pence et al., 2009). Given the varied manifestations of play along the child-teacher control continuum, further investigation remains imperative to elucidate the precise frequencies of inferential talk occurrences and the patterns of such discourse across different play typologies.

## 3 Verbal interactions between teachers and children within preschool play settings

From a Vygotskian sociocultural perspective, play functions as a dialogic medium in which children’s learning unfolds within co-constructed imaginary scenarios, with language serving as a primary semiotic tool for behavioral planning and self-regulation (Fleer, 2010; Hakkarainen, 2008). As play becomes more socially mediated, linguistic complexity escalates through role negotiation and rule-based discourse (Vygotsky, 1967). Play-based interactions facilitate more dynamic and cognitively rich verbal exchanges (Hadley and Dickinson, 2019; Hirsh-Pasek et al., 2008, 2015), particularly in pretend play, which offers unique opportunities for abstract conceptual exploration (Katz, 2001; Pellegrini, 1985). Such interactions are often typified by role allocation (e.g., “Who will be the wizard?”), narrative progression (e.g., “What spell should the wizard cast next?”), and fictional elaboration (e.g., “This flower is our enchanted microphone.”). Dramatic play settings enable teacher-facilitated scaffolding through play-embedded discourse (Meacham et al., 2013), promoting bidirectional, cognitively rich exchanges. Similarly, narrative-based play (e.g., picture-enhanced scenarios) fosters shared dialogic meaning-making (Lee and Wright, 2017). Empirical evidence also highlights that during symbolic play with constructive materials, teachers employ significantly more cognitively demanding speech (Gregory et al., 2003; Cohen and Emmons, 2016), characterized by a high frequency of open-ended questions (Liu, 2016) (e.g., “How can we improve the tower’s stability?”), guided problem-solving prompts (e.g., “Try using a triangular base.”) as well as spatial language modeling (e.g., “Position this block atop these shapes.”), which reinforces children’s spatial reasoning and domain-specific vocabulary acquisition (Hedge and Cohrssen, 2019).

Teachers often assume the role of language facilitators by extending discourse topics based on children’s immediate interests (Cabell et al., 2013; Krizek, 2011). When pedagogical questioning aligns with child-initiated play themes, both response rates and linguistic complexity surpass those observed during teacher-directed interactions (de Rivera et al., 2005; Meacham et al., 2015). Educators may further embed themselves in play narratives through role-consistent pretend speech, thereby modeling advanced language use (Gest et al., 2006; Kontos, 1999). Notably, play frameworks theoretically compensate for environmental constraints (Katz, 2001; Pellegrini, 1985), creating opportunities for cognitively demanding linguistic exchanges (Gest et al., 2006; Tompkins et al., 2013). However, existing research predominantly focuses on free play contexts, leaving other play modalities under examined. Given the dynamic power distribution between teachers and children across educational settings, verbal interaction patterns in diverse preschool play types remain insufficiently explored. Building on the play continuum model (Pyle and Danniels, 2016), it might be that free play naturally elicits open-ended discourse; inquiry play (teacher-following-child) necessitates predictive scaffolding, potentially triggering hypothetical reasoning; collaboratively designed play requires joint decision-making, fostering egalitarian negotiation; playful learning implicitly integrates learning objectives, facilitating guided dialogue; learning through games establish conditions for systematic explanations. To address these gaps, this study employs a tripartite framework—free play, guided play, and teacher-directed play—to analyze inferential talk patterns as indicators of high-quality dialogue and examine bidirectional interaction dynamics at the micro-linguistic level.

## 4 The current study

With particular attention to the control continuum governing adult-child participation during play, current scholarship has converged on a tripartite typology of pedagogical play: free play, guided play, and teacher-directed play (Pyle et al., 2020). Framed by this conceptual taxonomy and contextualized within the worldwide curricular reorientation toward play-based early childhood education, the present study systematically explore both the distinctive properties and reciprocal influence mechanisms characterizing these interactions across play typologies. We specifically analyzed teacher-child inferential discourse, as well as their conversational sequences and linguistic dyads. Our investigation is guided by two principal research inquiries:

(1) What context-specific configurations distinguish the developmental sophistication of inferential language employed by educators and preschoolers across different play types?
(2) Through what interactive patterns do the mutually constitutive dynamics of pedagogical inferential talk become instantiated within discrete play contexts?

## 5 Methods

### 5.1 Participants

This study centers on naturally occurring teacher-child verbal exchanges during preschool play activities, analyzing spontaneously generated linguistic data. The study utilizes authentic interaction records from the institutional initiated Children’s Social Interaction Corpus (CSIC). The CSIC project systematically documents naturalistic teacher-child dyadic interactions and peer communicative exchanges across 90 classrooms in 21 public preschools^2^ (representing a socioeconomically advanced coastal region in Mainland China). Data collection spanned complete half-day educational routines. The study reported in this paper implemented a stratified random sampling approach across three phases: (1) initial identification of 332 naturalistic play episodes from the complete teacher corpus, (2) proportional random selection of 18 representative episodes from each play category, yielding a final analytical sample of 54 video-recorded interactions, and (3) inclusion of all observable teachers and children within these episodes as study participants.

In total, the 54 play episodes analyzed in this study involved 41 certified early childhood teachers recruited from 16 public preschools. All teachers served as the lead teacher in their respective classrooms. The sample was predominantly female (97.56%), with only one male teacher. The participants had a mean age of 30.15 years (*SD* = 5.18). In terms of professional experience in early childhood education, the teachers reported an average of 7.41 years of teaching experience (*SD* = 4.99, range = 2–19 years). The highest academic qualifications among participants were distributed as follows: a majority held a bachelor’s degree (85.37%, *n* = 35), while a minority held either a postgraduate (9.76%, *n* = 4) or an associate degree (4.88%, *n* = 2). All teachers were Chinese, possessed a valid preschool teaching certification, and used Mandarin Chinese as the medium of instruction.

The participating children in this study were 637 children from the classrooms of 41 teachers. Their age distribution was as follows: 36.58% (*n* = 233) were 3–4-year-olds, 34.69% (*n* = 221) were 4–5-year-olds, and 28.73% (*n* = 183) were 5–6-year-olds. Gender distribution was relatively balanced, with boys comprising 49.14% (*n* = 313) and girls 50.86% (*n* = 324). Investigation into household annual income revealed diverse socioeconomic backgrounds: the vast majority (90.58%) were from families earning CNY 150,000 or more. Specifically, the largest proportion (44.74%) reported an income of CNY 150,000–300,000 (inclusive), 35.48% reported CNY 300,000–450,000 (inclusive), and 10.36% reported over CNY 450,000. Meanwhile, 9.42% of the children were from families earning below CNY 150,000. All children were Chinese, and Mandarin Chinese was the language of communication in the preschool.

### 5.2 Data collection

Ethical approval for this study was obtained from the Institutional Review Board (IRB). The research employed unobtrusive video recording to capture naturalistic teacher-child interactions during standard half-day preschool routines. Prior to data collection, formal communication were randomly established with multiple public preschools. The research protocol was submitted, discussed in detail, and written institutional consent was secured from each preschool’s administration. Eligible teachers and parents of children in their classroom were identified from consenting preschools when a comprehensive informed consent process was implemented. Participants were informed of the voluntary nature of their involvement and their right to pause or withdraw from the study at any time if they experienced discomfort. Data collection commenced only after all consent forms had been returned.

Filming schedules were coordinated with participating teachers in advance. Targeted observations focused on representative half-day instructional blocks. These observation sessions were exclusively scheduled during standard curricular days to ensure ecological validity. Each participating teacher was equipped with a professional-grade lapel microphone to capture clear audio dialogues. Assigned dedicated videographers are all with advanced degree candidates specializing in early childhood education and completed standardized protocol training prior to conducting observations: (1) The observer shall be proficient in fitting, calibrating, and troubleshooting lavalier microphones to ensure clear and continuous audio capture, and familiar with both stationary and mobile camera techniques to minimize disruption. (2) All recording activities shall comply with institutional research ethics review requirements. During filming, the observer must avoid capturing identifiable facial information of non-participating students and uninvolved individuals. (3) Recording shall be restricted to pre-scheduled instructional days and half-day sessions. The observer must remain silent and stationary, refrain from participating, and avoid interrupting teacher–child interactions. Should unforeseen circumstances arise (e.g., a child’s emotional distress or equipment malfunction), recording shall be immediately suspended and the incident reported to the research team. (4) The classroom’s seating arrangement, lighting, and daily routine shall remain unaltered; white balance and audio calibration shall be performed prior to recording to ensure footage faithfully reflects the naturalistic activity context. (5) Upon completion of each session, all raw footage shall be immediately encrypted and transferred to the principal investigator within 24 h.

Each observational session was designed to last approximately 180 min (±15 min), typically starting around 8:30 a.m. and concluding after the children finished their lunch. This duration ensured comprehensive data collection while accommodating natural transitions between activities. Throughout the filming, videographers refrained from conversing with teachers or children and did not interfere with normal activities. Furthermore, to minimize potential discomfort among children caused by the presence of strangers and recording equipment, videographers visited the classrooms in advance for familiarization. Filming was immediately discontinued if any child exhibited unwillingness to continue.

### 5.3 Data selection

This study implemented a rigorous mixed-methods sampling strategy combining purposive and stratified random sampling techniques to ensure methodological validity. Grounded in the conceptual framework of the play continuum, this study applied a broad definition of “preschool play” to include any activities within the preschool’s daily schedule that are child-centered and offer a playful experience. This definition encompasses a spectrum of practices, from highly autonomous free play to activities intentionally designed by teachers to be playful, reflecting variations in child control and structural organization.

Using this definition as a guide, an expert with a PhD in early childhood education, focusing on children’s play and development, provided specialized training to two researchers in the same field. Inter-rater agreement for identifying play episodes from six training videos reached 95.45%. The specific procedure consisted of the following steps: Each researcher independently watched every half-day video recording to identify all play episodes. These episodes were then named based on play themes and settings, and recorded their durations (e.g., “Indoor Obstacle Course Game—22 min,” “Outdoor Biking Game—37 min”). Each identified episode had to represent a complete activity sequence, encompassing the preparation phase, core implementation period, and conclusion. Coding was considered consistent if the raters’ timestamps for the start and end of an episode were within a 2-min window. Any disagreements were resolved by the training expert, who independently reviewed the disputed video segments and redefined their temporal and activity boundaries based on the definition above. The expert’s ruling was final for data selection, and the initial researchers were not involved. Subsequently, the two researchers identified play episodes across all classroom videos in the CSIC project, achieving an inter-rater reliability of 96.77%. Any remaining discrepancies were resolved by the expert in accordance with the procedure described above. This process yielded 364 independent play episodes, which were systematically identified and extracted from the complete half-day video recordings of the 90 participating classrooms in the CSIC project.

To maintain methodological alignment with our core research focus on inferential language exchanges, three additional exclusion criteria were applied: (1) episodes featuring purely observational teacher involvement without interaction, (2) interactions dominated by caregiving discourse (including but not limited to hydration management, toileting assistance, and clothing-related routines); and (3) episodes involving only peer interaction among children without teacher participation. Based on the aforementioned criteria, a primary researcher screened the 364 play episodes for eligibility, resulting in 332 episodes that met all study criteria. To ensure reliability, 20% of the episodes (*n* = 73) were randomly selected for double-coding by a second researcher, a proportion that aligns with the standards recommended by Syed and Nelson (2015) for narrative data coding reliability. This coding yielded a Cohen’s kappa of 0.936, indicating excellent inter-rater reliability.

The qualified episodes were first classified into three theoretically distinct play modalities: free play, guided play, and teacher-directed play. For each type of play activity, 6 representative episodes were randomly selected from each of the three developmental levels: Junior Class (3–4 years old), Middle Class (4–5 years old), and Senior Class (5–6 years old). This process generated our final analytical sample of 54 video-recorded episodes, with mean durations of 35 min (*SD* = 10.96) per recorded interaction.

### 5.4 Coding scheme

#### 5.4.1 Coding of play types

Through observational research, Pyle and Danniels (2016) conceptualized play as a continuum spanning varying degrees of child autonomy and teacher direction. This framework offers a valuable means to seamlessly integrate academic skill objectives with children’s play-based needs, while also supplying a more comprehensive and tangible conceptual model for capturing the complex and dynamic manifestations of play in preschool environments.

Although the original continuum includes five distinct types of play, the empirical study result underscores the current pedagogical predominance of play types with tripartite taxonomy (child-initiated free play, adult-scaffolded guided play, and teacher-directed play) documented in recent scholarly work (Pyle et al., 2020). Using this classification system, the present study further coded the 332 valid play episodes into these three categories. The analytical framework employs complete play episodes as discrete coding units. Operational definitions and illustrative examples for each play modality comprehensively as follows:

(1) Free play: Emphasizes child-initiated and child-directed open-ended activities where children freely choose materials, play partners, etc. Teachers provide certain responses to children’s play needs or interests but do not plan or design the play. For example: During area time, a child asked a peer building with blocks what he was making. Hearing it was a spinning top, the child suggested: “I’ll build one too and we can compete.” The teacher also asked to join. When the teacher’s top would not spin, the children offered advice like, “It needs a square bottom,” or, “There’s too much at the bottom.” As excitement grew, more children built tops and joined in. Noticing their engagement, the teacher provided more blocks but did not guide how they built or competed.
(2) Guided play: Establishes a bi-directional negotiation mechanism. Teachers construct play scenarios based on specific goals and employ participatory guidance strategies during play, while sharing decision-making authority with children. Children have the right to freely choose play materials and methods within defined parameters. The ultimate goals and content of the play exhibit flexibility and are adjusted in real-time based on children’s actual participation. For example: The teacher organized a domino activity focused on “understanding dominoes,” having children form groups of 4–6 and providing paper, colored pens, and domino sets for collaborative assembly. Early on, some groups disagreed on what shape to build. The teacher facilitated by summarizing differing opinions and asking how to resolve them. Children suggested voting or rock-paper-scissors, eventually using the latter to choose a pentagon in one group; others picked hearts or parallelograms. When a child proposed rewarding the fastest group with stickers, the teacher adapted by launching a domino assembly competition. This increased engagement as children raced to complete and topple their domino shapes.
(3) Teacher-directed play: Refers to teachers creating game environments related to curriculum content in accordance with predefined course objectives and requirements when designing preparatory lesson plans. Teachers meticulously design and organize teaching activities by incorporating game elements (e.g., game tones, role-playing, competitive games) and game methods (e.g., finger games), with rules preset and implementation monitored by the teacher. For example: Focusing on the objective of “learning hosting and guest etiquette,” the teacher created a birthday party role-play. Using props like cake, pizza, and plates, she announced: “Our baby is having a birthday—I invite all little guests to our home!” The children eagerly agreed to join. She then prompted: “What should we prepare when inviting guests?” to encourage thinking about host etiquette. Three children acted as “guests” visiting her “home,” while the rest observed. After the performance, the audience gave feedback. The teacher concluded by guiding a discussion on how to be a good host and guest.

A researcher coded the play types for all episodes. The coding results revealed that, of the 332 valid play episodes, 43.98% (*n* = 146) were coded as free play, with 27.40% from junior, 36.30% from middle, and 36.30% from senior classes. Guided play accounted for 36.45% (*n* = 121), distributed as 35.54% (junior), 38.84% (middle), and 25.62% (senior). Teacher-directed play constituted 19.58% (*n* = 65), comprising 16.92% (junior), 44.62% (middle), and 38.46% (senior) classes. To ensure coding consistency, 20% (*n* = 67) of the episodes were randomly selected for double-coding by another researcher, resulting in a Cohen’s kappa of 0.941, which indicates excellent inter-rater agreement.

#### 5.4.2 Messages

Verbal utterances and meaningful nonverbal behaviors between teachers and children were transcribed from 54 play session videos, forming textual corpora. These corpora were then segmented into semantic units following systemic functional linguistics (SFL) standards. Within functional grammar frameworks, the message serves as the fundamental semantic unit. According to Halliday and Matthiessen (2004), a message consists of exactly one explicit or implicit subject and one verb. A single sentence may contain multiple messages. For example, the utterance “You two put it in the box and play together” comprises two messages: “You two put it in the box” and “[You two] play together.” Body language that contains clear semantic meaning in interactions was also marked in the transcription, such as “nodding” to indicate an agreement, which stands as an independent message. A total of 37,920 messages were divided and input into an Excel spreadsheet for coding.

#### 5.4.3 Coding of inferential talk

Building upon Cognitive Distancing Theory, Blank et al. (1978) further subdivided inferential language into a continuum comprising two tiers and four distinct levels. According to their conceptualization, Level 1 (Matching Perception) entails activities such as naming or counting objects, while Level 2 (Integration of Perception) involves describing perceptual features. These first two levels are considered literal because they focus on the immediate environment and require little to no inference from the communicative participants. In contrast, the higher levels are inferential: Level 3 (Inferring about Perception) involves explaining specific viewpoints, and Level 4 (Reasoning about Perception) entails predicting future events, establishing causal connections, and similar higher-order reasoning. This framework of four mutually exclusive and collectively exhaustive levels categorizes language based on increasing cognitive demand.

The scheme has been widely employed in studies of adult-child verbal interactions (e.g., Hammett et al., 2003; Sorsby and Martlew, 1991; Tompkins et al., 2013; Zucker et al., 2010) and has undergone iterative refinements through empirical application. For this study, Tompkins et al. (2013) coding scheme served as the primary reference, with contextual adaptations informed by Liu (2023) work on Chinese early childhood settings. Revisions included minor modifications to specification items, such as the addition of Item 6 at Level 1: “Repeating a simple word or sentence” (see Table 1 for the complete coding scheme).

**Table 1:** Summary of sequential analysis codes and definitions.

Utterance	Level	Description	Examples
Literal utterance	Level 1 (T1/C1)^1^ matching perception	1. Labeling objects.	1.1 What is this?
		2. Locating objects.	1.2 Where is the mud board?
		3. Noticing objects/people.	1.3 Look at the castle I built
		4. Counting concrete objects/people.	1.4 How many little guests did I invite?
		5. A request for help with materials.	1.5 I want to use this block.
		6. Repeating a simple word or sentence.	1.6 Child: Need to build it firmly. Teacher: Yes, firmly.
	Level 2 (T2/C2) integration of perception	1. Describing object characteristics-perceptual qualities.	2.1 What color is this?
		2. Describing story actions/events/scene.	2.2 They are building a home for the little calf.
		3. Imitating.	2.3 I am the king, I want to sit on the throne
		4. Recalling actions/events/scene.	2.4 We built two castles yesterday.
		5. Giving simple direction up to two steps.	2.5 Can you pick up the block?
		6. Possession.	2.6 I also have a Huarong Dao (puzzle).
Inferential utterance	Level 3 (T3/C3) inferring about perception	1. Sequence of past or future events.	3.1 I want to build a zip line.
		2. Point of view/cognition/feeling.	3.2 I think the sofa can be placed next to the door./Is the egg really round?/I’m so scared.
		3. Judgment.	3.3 Is this a potato you drew?
		4. Inference.	3.4 Child: This is a cow factory. Teacher: Oh, this building is for the little calf to live in?
		5. Compare similarities/differences.	3.5 What is different between these two?
		6. Generalize.	3.6 So it combines the colors of mom and dad, right?
	Level 4 (T4/C4) reasoning about perception	1. Define word meaning.	4.1 A milk cup is a milk carton.
		2. Define the function/purpose of an object or print unit.	4.2 This long grid is for building houses.
		3. Predict/hypothesiz.	4.3 What happens to water outside when it is very cold?
		4. Justify or explain a prediction, judgment, or inference.	4.4 You can try it, see if red plus green makes purple.
		5. Explain conditions that cause alternate outcomes.	4.5 Move the block back a bit, so there is more space here.
		6. Identify causes of occurrence/event.	4.6 Teacher: Why did it fall? Child: Because of the electric fan.
		7. Identify direct or indirect effects.	4.7 Teacher: What if the egg gets crushed? Child: The chick will get crushed to death.
		8. Distinguish between fact and fiction.	4.8 They are playing a game, it is not real.
		9. Formulate a solution.	4.9 Teacher: How can we make it not fall? Child: I think we need to cement it with concrete.
		10. Identify essential/non-essential characteristics.	4.10 The top and bottom faces of a cylinder are both circles.

^1^Utterances produced by teachers were coded as T1, T2, T3, or T4 corresponding to Levels 1 through 4, respectively. Child utterances were similarly coded as C1, C2, C3, or C4 based on the same level criteria.

The coding scheme was consistently applied to all verbal exchanges between teachers and children throughout the study. During the analysis, certain types of utterances were systematically excluded from coding, including caregiving-related topics (e.g., toileting, drinking, dressing) and consecutive repetitions by the same speaker. Utterances demonstrating no discernible cognitive abstraction—such as brief evaluative comments (e.g., “It is great,” “Wonderful”) or filler words—were uniformly classified as L0 (Level 0). Necessary non-verbal responses during interaction (e.g., nodding, shaking head…) were also coded as “L0.” For simple verbal responses (e.g., “Yes,” “OK”), coders conducted comprehensive contextual analysis considering paralinguistic cues (intonation patterns) and nonverbal signals (facial expressions) before assigning them to either L0 or the appropriate higher cognitive level (Levels 1–4).

Following this scheme, a primary researcher coded the teacher and child messages in all 54 play episodes. To ensure coding reliability, a second researcher independently coded a randomly selected 20% (*n* = 11) of the episodes. The inter-rater reliability for this coding, as measured by Cohen’s kappa, was 0.863, indicating a high level of agreement.

#### 5.4.4 Sequences of inferential talk

Following the coding process, continuous discourse exchanges between teachers and children focusing on the same topic were delineated as distinct teacher-child interaction events. (Notably, events consisting solely of child–child verbal exchanges without teacher participation were excluded from analysis as invalid cases.) Within each primary play segment, multiple discrete interaction events were identified through thematic transitions. The initiation of an interaction event was operationally defined as occurring when one participant introduced a new topic that subsequently elicited a response from their conversational partner. For analytical purposes, each continuous speech segment produced by an individual participant was considered a turn—operationally defined as vocalization beginning at speech onset and continuing until speech offset—with each turn potentially containing multiple discrete messages.

**Figure 1:**
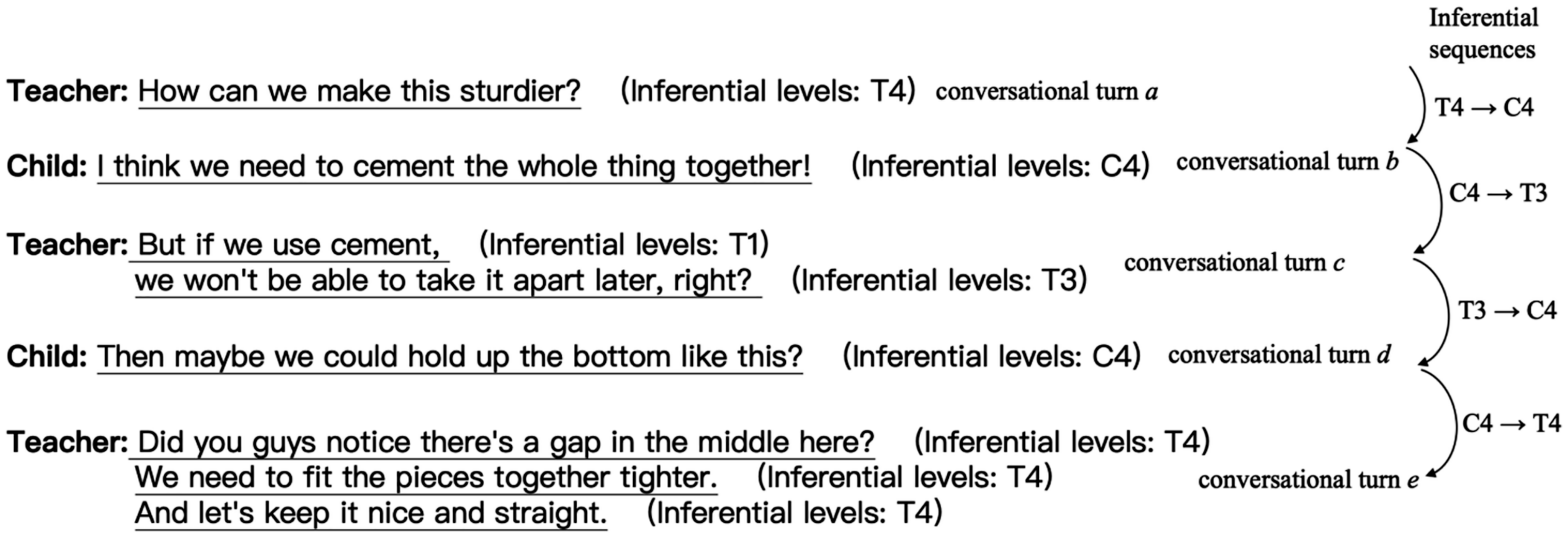
Exemplary approaches for constructing inferential sequences.

McCarthy (1992) established that paired utterances exhibit fundamental interdependence, where questions inherently anticipate answers and answers necessarily presuppose questions. In the context of teacher-child interactions, our analysis revealed that when a response from either participant prompted additional feedback, this subsequent exchange initiated a new paired sequence, thereby creating embedded structures of conversational turns. This framework allowed us to systematically identify two primary inferential sequence patterns within each interaction event: (1) teacher-initiated → child-response sequences, and (2) Child-initiated → Teacher-response sequences. Such sequence-based analysis proved particularly valuable for examining the reciprocal nature of inferential discourse between teachers and children, providing insights into the bidirectional dynamics of their conversational exchanges. The complete methodological specifications for sequence construction are presented in Figure 1.

This interaction event comprised 8 messages distributed across 5 conversational turns, which collectively formed 4 paired utterance sequences: *T4(a)* → *C4(b), C4(b)* → *T3(c), T3(c)* → *C4(d), and C4(d)* → *T4(e)*. In accordance with our coding protocol, when individual turns contained messages representing different inferential levels, the sequence construction was based on the highest-level inference present within that turn. This methodological approach ensured that each sequence accurately reflected the most cognitively demanding exchange within the interaction while maintaining the integrity of the conversational flow.

### 5.5 Data analysis

The study employed distinct analytical methods to address the two research questions: For Research Question 1, examining teacher-child inferential talk levels across different play types, we conducted descriptive statistics and analysis of variance (ANOVA) using SPSS 26.0. This quantitative approach allowed for systematic comparison of discourse patterns across play contexts. For Research Question 2, investigating interaction dynamics, a two-stage analytical process was implemented. First, we performed Lag Sequential Analysis (LSA) using GSEQ software (version 5.1) to assess transition probabilities and determine the statistical significance of behavioral sequence patterns (Sackett, 1978). This well-established method, widely adopted in psychological and educational research (Justice et al., 2013; Shao et al., 2019; Yang et al., 2016), enabled precise examination of interaction sequences. Subsequently, we conducted comparative analyses of inferential sequences using SPSS 26.0, specifically contrasting teacher-initiated → child-response sequences with Childinitiated→Teacher-response sequences. These analyses revealed significant differences that illuminated the bidirectional nature of teacher-child interaction dynamics in naturalistic settings.

## 6 Results

### 6.1 Level of inferential talk across play types

The 54 play session videos yielded 3,354 eligible teacher-child interaction events, containing 33,281 valid messages. Among these: Teachers produced 22,021 valid messages, accounting for 66.17% of total messages. Children produced 11,260 valid messages, accounting for 33.83% of total messages. This indicates teachers generated approximately twice the volume of children’s messages during interactions. Results of inferential talk levels across play types are presented below.

#### 6.1.1 Descriptive and variance analyses of the inferential level of teachers’ language

Welch’s ANOVA revealed significant differences among the inferential levels of teacher language (*p* < 0.001). The Games-Howell *post-hoc* test (Table 2) showed that Level 3 had the highest number of messages (*n* = 8,613), significantly exceeding Level 0 (*p* = 0.018), Level 1 (*p* = 0.024), and Level 4 (*p* = 0.025). This was followed by Level 2 (*n* = 6,307), which also had significantly more messages than Level 0 (*p* = 0.013), Level 1 (*p* = 0.018), and Level 4 (*p* = 0.023). Next were Level 4 (*n* = 3,415) and Level 1 (*n* = 2,929). Messages with no discernible abstraction were the least frequent (*n* = 757) and, being analytically uninformative, were excluded from subsequent analysis.

**Table 2:** Variance analyses of the inferential level of teachers’ language.

(I) inferential levels	Messages	(J) inferential levels	Mean difference (I-J)	Std. error	SIG.
Level 0	757	Level 1	−724.667*	70.858	0.016
		Level 2	−1850.667*	131.887	0.013
		Level 3	−2619.333*	208.347	0.018
		Level 4	−886.667*	87.764	0.020
Level 1	2,929	Level 2	−1126.000*	146.188	0.018
		Level 3	−1894.667*	217.681	0.024
		Level 4	−162.000	108.070	0.613
Level 2	6,307	Level 3	−768.667	244.455	0.166
		Level 4	964.000*	155.089	0.023
Level 3	8,613	Level 4	1732.667*	223.756	0.025

“*” indicates a significant difference at the 0.05 level.

The Kruskal-Wallis test (Table 3) revealed significant differences in message counts across play types for Level 1 (*p* = 0.01), Level 2 (*p* = 0.02), and Level 3 (*p* = 0.047). *Post hoc* pairwise comparisons showed that: for Level 1, messages were significantly more frequent in teacher-directed play than in guided play; for Level 2, counts in teacher-directed play were higher than in both free play and guided play, with significant differences among all three formats; and for Level 3, teacher-directed play contained more messages than free play.

**Table 3:** Variance analyses of the inferential level of teachers’ language across play types.

Inferential levels	Play types	Numbers	*M*	SD	SIG.	Pairwise comparison
Level 0	FP	18	15.11	11.406	0.084	
	GP	18	10.44	6.715		
	TDP	18	16.50	10.478		
Level 1	FP	18	56.39	20.425	0.010	GP < TDP
	GP	18	39.50	27.690		
	TDP	18	66.83	32.702		
Level 2	FP	18	101.50	31.554	0.020	FP < TDP GP < TDP
	GP	18	109.17	40.207		
	TDP	18	139.72	51.027		
Level 3	FP	18	142.78	47.237	0.047	FP < TDP
	GP	18	141.28	91.005		
	TDP	18	194.44	81.023		
Level 4	FP	18	61.94	24.981	0.761	
	GP	18	64.56	48.979		
	TDP	18	63.22	40.779		

FP, Free Play; GP, Guided Play; TDP, Teacher-directed Play.

#### 6.1.2 Descriptive and variance analyses of the inferential level of children’s language

Welch’s ANOVA indicated significant differences across the inferential levels of child language (*p* < 0.001). Post hoc Games-Howell tests (Table 4) showed that Level 3 had the most messages (*n* = 3,797), significantly more than Level 0 (*p* = 0.014), Level 1 (*p* = 0.006), and Level 4 (*p* = 0.001). Level 2 followed (*n* = 3,012), with a count significantly greater than that of Level 4 (*p* = 0.007). The least frequent were Level 1 (*n* = 1,666) and Level 4 (*n* = 886). Messages lacking discernible abstraction were excluded from further analysis as they were not analytically meaningful.

**Table 4:** Variance analyses of the inferential level of children’s language.

(I) inferential levels	Messages	(J) inferential levels	Mean difference (I-J)	Std. error	SIG.
Level 0	1899	Level 1	77.333	136.043	0.986
		Level 2	−371.000	136.043	0.194
		Level 3	−633.000*	136.043	0.014
		Level 4	337.333	136.043	0.264
Level 1	1,616	Level 2	−448.333	136.043	0.091
		Level 3	−710.333*	136.043	0.006
		Level 4	260.000	136.043	0.493
Level 2	3,012	Level 3	−262.000	136.043	0.486
		Level 4	708.333*	136.043	0.007
Level 3	3,797	Level 4	970.333*	136.043	0.001

“*” indicates a significant difference at the 0.05 level.

The Kruskal-Wallis test (Table 5) showed a significant difference in Level 1 message counts across play types (*p* = 0.01). Post hoc pairwise comparisons indicated that Level 1 messages were significantly more frequent during free play than guided play.

**Table 5:** Variance analyses of the inferential level of children’s language across play types.

Inferential levels	Play types	Numbers	*M*	SD	SIG.	Pairwise comparison
Level 0	FP	18	35.61	18.144	0.322	
	GP	18	31.22	18.941		
	TDP	18	38.67	18.790		
Level 1	FP	18	36.11	12.829	0.028	GP < FP
	GP	18	22.17	16.114		
	TDP	18	34.28	27.890		
Level 2	FP	18	61.39	28.216	0.084	
	GP	18	46.06	32.443		
	TDP	18	59.89	31.710		
Level 3	FP	18	67.56	28.295	0.060	
	GP	18	58.94	40.976		
	TDP	18	84.44	42.409		
Level 4	FP	18	17.89	12.019	0.372	
	GP	18	17.83	21.153		
	TDP	18	13.50	10.924		

FP, Free Play; GP, Guided Play; TDP, Teacher-directed Play.

Within the valid messages in play session videos, teachers produced approximately twice the volume of children’s utterances (66.17% vs. 33.83%). Across both teacher and child language, Level 3 (inferential) messages were most frequent, significantly surpassing all other levels, while teacher-directed play consistently yielded higher counts of lower-level inferential language (Levels 1–2) than free and guided play. Notably, children’s Level 1 messages were more frequent during free play than guided play, suggesting that different play formats elicit distinct inferential language patterns from both teachers and children.

### 6.2 Bi-directional dynamic of teacher-child inferential talk across play types

#### 6.2.1 Lag sequential and variance analyses of teacher-initiated → child-response inferential sequences

This study employed lag sequential analysis to identify inferential sequences where teachers’ initiations at specific inferential levels triggered statistically significant child responses. Within each interaction event, teachers’ inferential levels served as given events and children’s inferential levels served as target events. Z-scores for sequence frequencies were calculated from behavior transition frequency tables, generating a residual table (Table 6). Sequences with *Z* > +1.96 (*p* < 0.05) indicated statistical significance. Differences in teacher-initiated → child-response sequences across play types were further analyzed using Kruskal-Wallis tests with *post hoc* comparisons (Table 7). Key findings follow:

**Table 6:** Lag sequential analyses of teacher-initiated → child-response inferential sequences.

ADJR	Target									
Given	C1		C2		C3		C4		C0	
T1	FP	*Z* = 15.06								
	GP	*Z* = 15.22								
	TDP	*Z* = 16.38								
T2			FP	*Z* = 7.38					GP	*Z* = 6.44
			GP	*Z* = 10.05					TDP	*Z* = 5.14
			TDP	*Z* = 10.43						
T3					FP	*Z* = 10.08				
					GP	*Z* = 11.51				
					TDP	*Z* = 16.05				
T4							FP	*Z* = 11.50	FP	*Z* = 3.33
							GP	*Z* = 11.05	GP	*Z* = 2.64
							TDP	*Z* = 12.99	TDP	*Z* = 2.54
T0			FP	*Z* = 2.73					TDP	*Z* = 3.11

FP, Free Play; GP, Guided Play; TDP, Teacher-directed Play.

**Table 7:** Variance analyses of teacher-initiated → child-response inferential sequences.

Inferential squences	Play types	Numbers	*M*	SD	SIG.	Pairwise comparison
T1 → C0	FP	18	4.67	3.308	0.011	GP < FP
	GP	18	1.61	2.173		
	TDP	18	3.44	3.914		
T1 → C2	FP	18	11.5	7.532	0.012	GP < FP
	GP	18	5.11	6.249		
	TDP	18	7.67	7.670		
T2 → C3	FP	18	18.72	10.289	0.010	FP < TDP GP < TDP
	GP	18	15.94	9.704		
	TDP	18	27.17	12.908		
T3 → C0	FP	18	48.06	28.335	0.049	GP < TDP
	GP	18	40.00	32.322		
	TDP	18	61.11	32.696		
T3 → C3	FP	18	96.17	39.518	0.003	FP < TDP GP < TDP
	GP	18	94.44	69.692		
	TDP	18	162.83	76.781		

FP, Free Play; GP, Guided Play; TDP, Teacher-directed Play.

As for Level 1. T1 → C1 sequences were significant (*Z* > +1.96) across all play types, indicating Level 1 teacher initiations most likely elicited Level 1 child responses. Besides, T1 → C0 and T1 → C2 sequences differed significantly between free play and guided play (*p* = 0.011; *p* = 0.012), with higher frequency in free play. As for Level 2, T2 → C2 sequences were significant (*Z* > +1.96) across all types, indicating Level 2 teacher initiations most likely elicited Level 2 child responses. And T2 → C3 sequences differed significantly across types (*p* = 0.01), with highest frequency in teacher-directed play. As for Level 3, T3 → C3 sequences were significant (*Z* > +1.96) across all types, indicating Level 3 teacher initiations most likely elicited Level 3 child responses. While, this sequences differed across types (*p* = 0.003), highest in teacher-directed play. Also, T3 → C0 sequences differed between guided play and teacher-directed play (*p* = 0.049), higher in teacher-directed play. As for Level 4, T4 → C4 sequences were significant (*Z* > +1.96) across all types, indicating Level 4 teacher initiations most likely elicited Level 4 child responses. There were no significant differences in other sequences.

#### 6.2.2 Lag sequential and variance analyses of child-initiated → teacher-response inferential sequences

Within each interaction event, child inferential levels served as given events and teacher levels as target events, yielding a residual table for child-initiated sequences (as shown in Table 8). Table 9 presents variance analyses across play types.

**Table 8:** Lag sequential analyses of Child-initiated → Teacher-response inferential sequences.

ADJR	Target									
Given	T1		T2		T3		T4		T0	
C1	FP	*Z* = 5.92								
	GP	*Z* = 6.4								
	TDP	*Z* = 9.1								
C2			FP	*Z* = 2.00					FP	*Z* = 2.45
			GP	*Z* = 3.45					GP	*Z* = 2.26
			TDP	*Z* = 4.87						
C3					FP	*Z* = 5.68				
					GP	*Z* = 6.88				
					TDP	*Z* = 10.75				
C4							FP	*Z* = 9.25		
							GP	*Z* = 8.92		
							TDP	*Z* = 10.31		
C0			FP	*Z* = 3.07					FP	*Z* = 3.48
			GP	*Z* = 3.92					GP	*Z* = 2.15
			TDP	*Z* = 8.31					TDP	*Z* = 1.25

FP, Free Play; GP, Guided Play; TDP, Teacher-directed Play.

**Table 9:** Variance analyses of Child-initiated → Teacher-response inferential sequences.

Inferential squences	Play types	Numbers	M	SD	SIG.	Pairwise comparison
C0 → T2	FP	18	11.72	11.473	0.017	FP < TDP
	GP	18	14.39	16.026		
	TDP	18	21.44	13.631		
C1 → T3	FP	18	38.28	19.081	0.031	GP < TDP
	GP	18	21.94	21.165		
	TDP	18	43.67	38.059		
C3 → T3	FP	18	89.33	42.604	0.004	FP < TDP GP < TDP
	GP	18	94.72	54.817		
	TDP	18	155.72	81.004		

FP, Free Play; GP, Guided Play; TDP, Teacher-directed Play.

As for Level 1, C1 → T1 sequences were significant (*Z* > +1.96) across all types, indicating Level 1 child initiations most likely elicited Level 1 teacher responses. Besides, C1 → T3 sequences differed significantly between guided play and teacher-directed play (*p* = 0.031), with higher frequency in teacher-directed play. As for Level 2, C2 → T2 sequences were significant (*Z* > +1.96) across all types, indicating Level 2 child initiations most likely elicited Level 2 teacher responses. There were no significant differences in other sequences. As for Level 3, C3 → T3 sequences were significant (*Z* > +1.96) across all types. Indicating Level 3 child initiations most likely elicited Level 3 teacher responses. Specifically, C3 → T3 sequences differed significantly across types (*p* = 0.004), with highest frequency in teacher-directed play. As for Level 4, C4 → T4 sequences were significant (*Z* > +1.96) across all types, indicating Level 4 child initiations most likely elicited Level 4 teacher responses. There were no significant differences in other sequences.

Lag sequential analysis revealed a consistent level-matched pattern across all three play types: teacher initiations at each inferential level (Levels 1–4) most reliably elicited same-level child responses, and vice versa, confirming a strong bi-directional symmetry in inferential talk. Play type significantly moderated the frequency of certain cross-level sequences: teacher-directed play yielded the highest frequencies of higher-order inferential exchanges (e.g., T2 → C3, T3 → C3, C3 → T3), whereas free play showed comparatively higher frequencies of lower-level sequences (e.g., T1 → C0, T1 → C2). These findings collectively demonstrated that the play context shapes not only the inferential level at which participants initiate talk but also the extent to which they match or extend each other’s inferential level, underscoring the role of structured play in promoting higher-order inferential discourse.

## 7 Discussion

### 7.1 Characteristics of the level of inferential talk across play types

This study employs quantitative analysis to uncover features of inferential language in teacher-child interactions across diverse play contexts. Overall, Level 3 messages represented the highest proportion of teachers’ inferential language, followed by Level 2, with children’s inferential language exhibiting the same pattern. These findings partially align with those of Zucker et al. (2010), who observed during teacher-child book reading that children’s Level 3 inferential language peaked, followed by Level 1. This suggests that play-based interactions hold comparable potential to book reading in eliciting inferential dialogue and may even hold a slight advantage in promoting higher levels of inferential reasoning in children. However, certain discrepancies emerge when compared with Tompkins et al. (2013), who reported during small-group play that teachers’ Level 3 language was most frequent (followed by Level 1), while children’s responses were dominated by Level 1, followed by Level 3. This divergence may be attributed to the composite effects of activity types along the play continuum. Unlike narrowly defined small-group tasks, the present study encompasses a broader spectrum of guided and teacher-facilitated multimodal play contexts beyond free play, where collaborative and structurally embedded participation processes may better stimulate high-level inferential dialogue in teacher–child interaction.

Specifically, within-group comparisons revealed a consistent pattern of inferential language levels across all three play activities: for both teachers and children, Level 3 messages were the most frequent, followed by Level 2. This suggests that, regardless of play format, teachers prioritized language strategies that challenged children to move beyond superficial information and engage in reasoning and connection-making (Level 3), while also providing supportive scaffolding that links specific pieces of information (Level 2) to facilitate learning. Correspondingly, children consistently produced the highest proportion of Level 3 inferences, with Level 2 being the second most frequent across all play settings. These results not only reflect children’s inherent cognitive capacity and linguistic potential for higher-order thinking within diverse play-based environments (Auger et al., 2007; Lockhart, 2010; Hao and Hu, 2021; Tompkins et al., 2013), but also underscore the efficacy of teachers’ strategic use of high-level questioning and guidance in eliciting sophisticated language and thought. Children’s active engagement in producing high-level inferential language further affirms the role of play as a productive scaffold within the zone of proximal development (Vygotsky, 1967). By synergistically employing Levels 3 and 2 language, teachers created dialogic spaces that bridged current abilities and targeted higher cognitive potentials. This coordinated alignment in the distribution of inferential talk between teachers and children forms a generative interactive foundation within play contexts, serving as a key mechanism for promoting cognitive and linguistic growth.

Between-group comparisons revealed that teacher-directed play yielded significant advantages in promoting inferential dialogue between teachers and children. In this play context, teachers produced significantly more Level 1 messages than in guided play, more Level 2 messages than in either of the other two settings, and more Level 3 messages than in free play. This finding contrasts with previous research advocating for a balanced mix of guided and free play as optimal for child development (e.g., Sylva et al., 2004). Teacher-directed play effectively merges clear learning objectives with open-ended interaction, using rules and tasks to scaffold higher-order thinking while still allowing enough autonomy to encourage creative expression. This model of “structured exploration” offers a more productive environment for inferential language use than completely open free play, while also being more responsive to young children’s interests and developmental needs than traditional whole-class instruction.

Typically organized around specific learning goals, based on the findings of the study reported here, teacher-directed play incorporates playful tasks (e.g., a “shape sorting challenge”) that pose clear questions (“How can these shapes be grouped?”), prompting children to engage in comparison, categorization, and generalization. Teachers often intentionally embed key concepts and vocabulary while employing strategic questioning to promote substantive dialogue. For instance, they may pose hypotheticals grounded in descriptions of narrative play scenarios (“If you were to move a mountain, what tools would you need?”) to elicit prediction and planning, or extending from intuitive responses to explanatory questions (“Why does placing two bananas here represent the number 2?”) to lead logical argumentation. From the perspective of dialogic teaching—an instructional approach employing structured talk to stimulate thinking, advance understanding, and construct and evaluate arguments (Alexander, 2017)—such questioning functions not merely as information retrieval but as a medium for meaning negotiation, fostering children’s metacognition and prompting them to articulate the logic underlying their actions, thereby transforming unconscious manipulation into deliberate thought (Benedict-Chambers et al., 2017). It further guides children to connect fragmented experiences and construct causal chains (Caño and Sanz, 2025). Such strategic questioning often unfolds within sequences that span multiple cognitive levels, progressing from recall and judgment to reasoning and evaluation, for instance, from descriptive questions about “what is observed,” through comparative and hypothetical questions about “why it occurred,” to predictive and problem-solving questions about “how it might be improved,” thereby guiding children to decompose problems and engage in increasingly rigorous logical reasoning.

Teachers also maintain vigilant attention to reasoning gaps, offering corrective feedback (e.g., “You said UV rays are strongest here. Why, then, did the UV-sensitive bead become lighter?”) to induce reflection, and employing open-ended counter-questions (e.g., “I want to put all these vegetables together, and how should we eat them?”) to extend thinking. This suggests that highly structured, purposeful teacher-led play embeds a deliberate “talk” architecture, characterized by the sustained Initiation–Response–Feedback (IRF) interactional pattern (Avis and Hesmondhalgh, 2025), wherein teachers’ timely feedback triggers cognitive conflict, prompting children to recognize contradictions and reorganize existing cognitive schemas. Although teachers direct the overall trajectory of activities, children retain operational autonomy when selecting strategies, solving problems, and expressing ideas within supportive structures. The use of open-ended counter-questions reflects the core principle of scaffolded instruction: children preserve agency in exploration and expression even under teachers’ guidance (Lepola et al., 2026; Wasik et al., 2022). In this respect, teacher-led play holds distinctive and indispensable educational value in organizing and facilitating cognitively rich dialogue between teachers and young children.

### 7.2 Interaction features of teacher-child inferential talk across play types

Analysis of inferential talk levels between teachers and children captures only unilateral linguistic characteristics of each participant. Beyond this, the current study also aimed to uncover how teachers and children engage in cognitive interaction through inferential talk across different play contexts by examining linguistic sequences from a micro-linguistic perspective. Lag sequential analysis revealed significant consistency across all three play types: the level of children’s responses corresponded to the level of teachers’ initiating utterances, and similarly, the level of teachers’ responses aligned with that of children’s initiating utterances. These findings are consistent with previous research in book reading and instructional settings (Chen and Liang, 2017; Danis et al., 2000; Fei et al., 2020; Jin, 2014; Zucker et al., 2010).

This coherence reflects the inherent cohesion and sequential nature of dialogue. Specifically, within adjacency pairs, the first pair-part typically shapes the subsequent response, as speakers naturally adjust their replies in accordance with the content and tone of the preceding turn (Muhammad, 2017). Systemic functional linguistics further posits that language users flexibly adapt their linguistic behavior to contextual shifts, aligning it with interlocutors’ stages of cognitive development and thereby facilitating cognitive growth (Halliday and Matthiessen, 2004). This phenomenon further reveals that language acquisition is not an isolated individual process but a critical mechanism through which children, via imitation, adjustment, and internalization within the “other” mirror constructed by adults, progressively accomplish the transformation from external social activity to internal mental function. Drawing on Vygotsky’s theoretical account of human social interaction, children’s higher mental functions first manifest as interpsychological activity before being internalized as individual modes of thought (Vygotsky, 1981). Dialogic interaction furnishes children with an array of “psychological tools,” enabling the socialization of cognitive development through language as its primary medium (Vygotsky, 1986). The present study finds that such dynamic, reciprocal linguistic adjustment occurs more frequently in guided play and teacher-led play. Informed by these theoretical perspectives, the scaffolding role of the teacher in such play contexts is particularly salient, that is, in pursuit of specified educational objectives, teachers consciously calibrate the cognitive register of dialogue, such as deploying highly inferential language to challenge children or moderately complex language to provide support, thereby seeking to draw discourse into children’s zone of proximal development (ZPD) for language use. In response, children mobilize cognitive resources for integration and reasoning, sustaining or advancing the dialogic exchange.

It has been demonstrated that adults’ language proficiency shapes children’s response patterns, with children’s linguistic output, in terms of vocabulary, sentence length, and syntactic structure, closely mirroring adult input (Justice et al., 2013; Zucker et al., 2021). Adults also typically adjust their linguistic complexity (e.g., through child-directed speech), prompting children to match adult-initiated levels through effortful engagement and thereby fostering the development of conversational competence (Elmlinger et al., 2025; Malloy et al., 2022). The bidirectional alignment in dialogic sequences identified in this study indicates that adults do not engage in unidirectional transmission; rather, they flexibly calibrate their contributions in response to children’s self-initiated or reactive reasoning levels, whether high-level conjectures or low-level descriptions, sustaining dialogue within a mutually co-constructible cognitive zone. This further demonstrates that adult linguistic input creates conditions for children’s language processing and the logical construction and reorganization of thought, while children’s agency and orientation in language acquisition render adult input efficacious; the two mutually condition each other, jointly accounting for the high degree of alignment between response and initiation levels. The specific manifestations of teacher–child cognitive interaction across different play types are reflected in the following respects:

#### 7.2.1 Free play: low-level reciprocity

The study found that free play exhibited significantly higher frequencies of T1 → C0 and T1 → C2 sequences compared to guided play. This suggests that when teachers used Level 1 initiations, children were more likely to respond with either no abstraction (C0) or a Level 2 inference. Level 1 language, which centers on cognitive matching and factual descriptions of immediate people or objects, appears to prompt responses that remain concrete or only moderately abstract. Furthermore, free play showed the lowest frequencies of T3 → C3 and C3 → T3 sequences among all three play types. This indicates that initiations involving Level 3 language whether from the teacher or the child were less likely to elicit a Level 3 response from the other party. Level 3 utterances require cognitive inference, such as connecting objects or events beyond the immediate spatiotemporal context (e.g., through displacement reference or past experiences) and often integrate subjective interpretation. The scarcity of mutual Level 3 exchanges reflects a disruption in sustained cognitive engagement and deeper dialogic reciprocity between teachers and children in free play settings.

In free play, children predominantly control the discourse, while teachers typically assume the role of communicative “followers” (Andrews, 2015). Unless children actively pose questions or express complex ideas, teachers generally avoid initiating high-level dialogue so as not to undermine children’s autonomy. When children are deeply immersed in their own play scenarios and trains of thought, producing high-level responses would require them to interrupt their ongoing activity to engage in deep cognitive processes, such as recalling, associating, reasoning, or expressing subjective ideas, potentially disrupting their state of flow. Moreover, topics of conversation in this context shift rapidly according to children’s fluctuating play interests (e.g., abruptly transitioning from “building blocks” to “dinosaurs fighting”), which makes it challenging for teachers to maintain a coherent cognitive thread. Dialogues often center on emotional expression and rule negotiation (e.g., Child: “I still want to play!” Teacher: “Then play a bit longer.”/Child: “He knocked mine down.” Teacher: “Maybe move a bit to the side.”) Rather than conceptual exploration. The use of materials is also relatively unstructured, limiting teachers’ ability to leverage specific objects (e.g., using “magnets attracting nails” to introduce the concept of magnetism) to deepen the discussion or scaffold higher-level exchanges.

Research indicates that teachers are able to enhance the learning experience during play by assuming roles such as commentators, co-players, questioners, or by demonstrating how to interact with materials (Fisher et al., 2013; Tsao, 2008; Weisberg et al., 2013). However, in free play contexts, teachers often lack sufficient insight into the specific games children are engaged in, making it difficult to comprehend the underlying intentions, narrative developments, or internal logic of each child’s play. Consequently, teachers tend to default to surface-level questions (e.g., “What are you doing?” or “What is this?”) rather than formulating prompts that elicit deeper reasoning. The need to attend rapidly to multiple children or small groups further fragments the interaction, leaving teachers with inadequate time to wait for, guide, or capitalize on opportunities for fostering profound dialogue.

#### 7.2.2 Guided play: matched-level engagement

Compared to the other two play types, guided play had the fewest occurrences of T1 → C0 and T3 → C0 sequences. This indicates that after teachers initiated at inferential Levels 1 or 3, children were less likely to respond with no discernible abstraction (C0). Meanwhile, matching sequences (T1 → C1, T2 → C2, T3 → C3, C1 → T1, C2 → T2, C3 → T3) also occurred significant in guided play. Together, these patterns suggest more effective cognitive matching in this context: a given level of teacher inferential language tended to elicit a corresponding level of abstraction in children’s responses.

Guided play occupies an intermediate position on the playful learning continuum (Pyle and Danniels, 2016), reflecting a relative balance between child autonomy and adult guidance (Weisberg et al., 2016). In this mode, both teachers and children rely on one another to sustain the flow of the game. Teachers typically possess a clear understanding of the progression and internal logic of play activities, allowing them to calibrate the complexity of their language in response to children’s immediate reactions, thereby scaffolding toward learning goals (Jensen et al., 2019; Weisberg et al., 2013; Zosh et al., 2018). At the same time, children retain a sense of agency and intrinsic motivation to continue playing (Zosh et al., 2018), demonstrating willingness to engage with the teachers’ reasoning within a shared narrative. This mutual alignment often results in cognitively matched responses. For instance, when a teacher poses a Level 3 challenge such as “turn it into a curved moon,” the child may produce a corresponding Level 3 response like “like a little boat.”

While preserving a certain degree of children’s autonomy in selecting play themes and determining how to play, teachers maintained a moderate density of intervention through targeted questioning. For instance, in a situation where congestion occurred at the entrance of a play area, the teacher identified the immediate dilemma and posed a Level 4 question: “Look, they are blocking the way. What should you say at this time?” This context-sensitive prompt encouraged children to reflect on the obstacle preventing their participation, making it more likely for them to generate a rule-based response at a corresponding level of abstraction, such as saying, “Please be considerate.” The teacher then employed conceptual reframing to semantically elevate the child’s utterance to Level 3: “Oh, so you mean, ‘Please don’t crowd’, right?” After receiving the child’s confirmation, the teacher introduced a Level 4 hypothetical scenario: “What if someone cuts in line?” This strategic follow-up effectively created cognitive conflict, ultimately leading the child to express a rule-based understanding at Level 4: “Please don’t cut in line.”

This interactive sequence demonstrates how the teacher translated an abstract social norm (“consideration”) into concrete behavioral guidance (“Please do not crowd”), thereby supporting the child’s cognitive transition from concrete to abstract thinking. Through a dialogic spiral of “question → response → follow-up,” the teacher guided the child through logically scaffolded reasoning, forming an optimized interaction pattern (T4 → C4 → T3 → C3 → T4 → C4) that enabled the child to actively construct social rule cognition. When the child produced an initial response, the teacher used scenario expansion to induce cognitive disequilibrium, ultimately elevating the child’s reasoning from situation-specific coping (Level 2) to generalized rule understanding and multi-factor problem-solving (Level 4). This example underscores the unique capacity of guided play to foster higher-order thinking (Ramani and Siegler, 2008; Siegler and Ramani, 2008, Siegler and Ramani, 2009). Effectively integrating child autonomy, inquiry-based exploration, and structured guidance, this approach retains the fundamental qualities of play while enriching cognitive dialogue and providing tailored linguistic scaffolding (Weisberg et al., 2013). Thus, guided play serves as a balanced pedagogical model that harmonizes developmental appropriateness with educational intentionality.

#### 7.2.3 Teacher-directed play: progressive-level scaffolding

Compared to the other two play types, teacher-directed play exhibited significantly higher frequencies of T3 → C3 and C3 → T3 sequences. This indicates that when either the teacher or the child initiated a Level 3 utterance, the other was more likely to respond at the same high inferential level. Furthermore, teacher-directed play also showed significantly higher frequencies of T2 → C3, C0 → T2, and C1 → T3 sequences, suggesting that responses often exceeded the cognitive level of the initial prompt, reflecting a greater degree of inferential escalation from both participants. The pre-designed and structured nature of teacher-directed play optimizes both the accessibility and the challenge of cognitive input. Teachers’ intentional scaffolding lowers the threshold for engaging in higher-order thinking, while clearly defined goals and rules continuously prompt children to elevate their reasoning. For example, a teacher’s Level 2 question grounded in concrete situations (“What are the characteristics of this toothpaste box?”) can elicit Level 3 comparisons and associations from children (“It has many faces, some long, some short,” “Like a rectangle, but not exactly,” “It has pointy corners, like the dice in my flying chess set”).

Moreover, guided by explicit learning objectives, teachers are skilled at transforming children’s perceptual labeling into conceptual thought. When a child produces a Level 1 identification (“This is a cake”), the teacher can elevate the dialogue to Level 3 to foster systematic thinking (“Is this cake a rectangular prism or a cube? How is it similar to or different from that rectangular prism or cube over there?”). Anchored in mathematical or social rules, teachers also sustain extended sequences of high-level verbal exchange. For instance, to deepen understanding of geometric properties, a teacher may maintain a Level 4 dialogue sequence—moving from causal questioning (“Why do we place the toothpaste box with its longer side down?”) to analogical explanation (“Because the larger surface area on the longer side makes it more stable”)—thereby sustaining a focused exploration of structural stability.

Teacher-directed play typically relies on a pre-structured “engine” designed to elevate children’s abstract thinking. This framework involves clearly defined cognitive logic embedded within play rules (e.g., classifying objects by geometric features), pre-configured exploratory dimensions through material combinations (e.g., differences in stacking or placing objects of varying structural shapes), and social cognition introduced through role assignments (e.g., stocker, architect). Throughout the process, teachers dynamically adjust the operation of this engine to maintain playfulness, flexibly modulating the abstraction level of their language in response to the complexity of children’s reactions. This approach demonstrates how teacher-directed play harnesses the mediating function of “cultural tools” through structured verbal interaction, transforming higher-order thinking from an incidental occurrence into an intentional developmental outcome. However, this format also carries potential risks: an excessive emphasis on elevating cognitive complexity may cause teachers to overlook each child’s actual state of readiness, thereby producing a structural scaffolding discontinuity in activity design and effectively severing the necessary pathway through which a child progress from “actual developmental level” to his/her “potential level” (Carranza-Pinedo and Diprossimo, 2025). When the support provided in teacher-organized activities exceeds the upper limit of children’s zone of proximal development (ZPD) and lacks the requisite intermediate steps, children are unable to achieve task goals or establish connections between new and prior knowledge, despite their efforts. This situation readily gives rise to learned helplessness induced by cognitive overload (Parrish et al., 2025) or even defensive avoidance triggered by the absence of emotional support (Lepola et al., 2004). For instance, abruptly introducing the distinction between two-dimensional and three-dimensional geometric figures without adequate contextual or experiential grounding may precipitate cognitive discontinuity in children. Such a phenomenon may manifest through behavioral cues such as head-shaking and blank expressions, or through simplified language output, as evidenced by a marked decline in the diversity or complexity of children’s responses (e.g., “It’s this one,” “They’re all the same,” “The square is flat”).

Overall, teacher-mediated play formats namely guided play and teacher-directed play demonstrate distinct advantages in fostering high-level inferential dialogue between teachers and children. Guided play relies on a “dialogue extension mechanism,” where teachers use immediate follow-up questions (e.g., “How did you come up with that solution?”) to elicit and make children’s thinking explicit. In contrast, teacher-directed play operates through a “situational anchoring mechanism” embedding cognitive and linguistic scaffolds within pre-designed play structures such as roles and rules to provide stable conceptual support. Due to its dual-oriented nature, guided play allows teachers to dynamically adjust the trajectory of children’s thinking based on their ongoing play behavior, resulting in a spiral progression within inferential sequences. Teacher-directed play, being more teacher-orchestrated, typically follows a pre-planned hierarchical structure that guides thinking in a stepwise manner.

Flexibly transitioning between these play formats in response to children’s real-time verbal performance—thereby leveraging their complementary strengths—may represent a promising direction for designing effective play-based learning environments in preschools. By conducting micro-level analysis of inferential talk levels and linguistic sequences across different play contexts, this study broadens the analytical scope of language socialization research. Future studies should further incorporate multimodal perspectives to holistically examine teacher-child verbal interactions within situated play contexts, with the aim of comprehensively understanding the pathways through which play supports language acquisition and cognitive development.

### 7.3 Limitations and implication

The present study has yielded meaningful findings, though certain limitations warrant acknowledgment. First, the sample was drawn from kindergartens in a single economically developed coastal province in mainland China and, despite encompassing diverse institutional types, teacher backgrounds, and class levels, may not fully represent inferential talk practices across the country. Future research should broaden the geographical scope to include regions of varying economic and cultural contexts, thereby strengthening external validity. Second, the study focused exclusively on teacher–child inferential language and did not systematically analyze peer inferential exchanges, which are equally significant for cognitive development. Incorporating peer interaction into the analytical framework in future research would yield a more comprehensive account of children’s language development.

Notwithstanding these limitations, the study provides important empirical evidence for understanding the characteristics and interactional patterns of teacher–child inferential talk across diverse play contexts. It extends the applicability of the play continuum concept within language socialization research by revealing the micro-linguistic mechanisms through which different play forms regulate teacher–child cognitive tension via the distribution of interactional control. The findings also offer concrete pedagogical guidance: teachers should adapt their language strategies to the play type, capitalizing on teachable moments and judiciously introducing higher-order topics in free play, leveraging the bidirectional interaction of guided play to achieve a spiral progression of logic across linguistic levels, and strategically pre-designing cognitive scaffolding in teacher-led play to avoid the language simplification that often accompanies children’s psychological withdrawal. Teachers are also advised to dynamically shift across play forms in response to children’s immediate linguistic feedback, thereby fully harnessing the distinctive potential of each play type in fostering inferential language development.

## 8 Conclusion

Based on the conceptual framework of the “play continuum,” this study examines teacher-child inferential talk across free play, guided play, and teacher-directed play, thereby offering a new perspective for understanding verbal interaction in diverse play-based settings. Departing from prior research focused on shared book reading or structured instruction, this study makes a distinct contribution by revealing how play types—particularly as defined by their degree of structural organization—shape both the level of inferential language used and the dynamics of teacher-child interaction. The findings indicate that the manner in which teachers structure play arrangements is a critical variable in fostering cognitive leaps in young children. While the low-pressure environment of free play allows inferential talk to emerge spontaneously, such talk often remains at the level of describing and linking immediate events. In contrast, when teachers intervene as co-inquirers in guided play or act as cognitive architects in teacher-directed play, the activity shifts from random exploration to a scaffolded process of cognitive construction. Teachers are possible to foster this process through several key strategies: such as posing sustained heuristic questions (e.g., “Why?” “What if?”) to elicit explanations and predictions; creating cognitive conflicts by introducing counter-evidence to prompt deeper reasoning (e.g., “This differs from what we thought. Why might that be?”); and/or offering metacognitive prompts to guide reflection on thinking itself (e.g., “Was our guess correct?”). Through such goal-oriented interactions, teachers provide the cognitive framework that helps children connect concrete actions with abstract understanding.

Thus, the central conclusion of this study is that when teachers actively structure or guide children’s play, they consciously construct and extend shared conversational topics, aiming to align with and elevate children’s current inferential reasoning toward the co-construction of more abstract meaning. Simultaneously, within this shared dialogic space, children driven by the need to sustain the play are able to maintain, revise, or even advance their meaning-making at this elevated level. Consequently, educational practice should shift focus from merely selecting play types to examining how teachers implement strategic, reasoning-focused dialogue across different play contexts, thereby transforming play into a dynamic environment for cultivating inferential skills. Theoretically, through micro-discourse analysis, this study extends Language Socialization Theory into informal learning contexts by positioning teacher guidance as a central mechanism that mediates between concrete scenarios and abstract thought. Future research should employ multimodal analysis to explore how speech, gesture, and object manipulation interact synergistically during play-based cognitive construction, offering a more robust empirical foundation for designing high-quality, playful learning environments.

## Data Availability

The original contributions presented in the study are included in the article/supplementary material, further inquiries can be directed to the corresponding author.

## Footnotes

1 Conversational turns are the fundamental units of dialogue, defined as an instance where one person speaks and is then responded to by another. More conversational turns signify that children are actively engaged in processing information, structuring their thoughts, and articulating ideas.

2 Chinese public preschools are institutions that provide early education and care to children aged 3–6.
